# Effects of Different Visual Flow Velocities on Psychophysiological Responses During Virtual Reality Cycling

**DOI:** 10.7759/cureus.62397

**Published:** 2024-06-14

**Authors:** Kyosuke Kawaguchi, Takefumi Moriuchi, Ryotaro Takita, Kyosuke Yoshimura, Ryo Kozu, Yorihide Yanagita, Tomoki Origuchi, Takashi Matsuo, Toshio Higashi

**Affiliations:** 1 Occupational Therapy Science, Nagasaki University Graduate School of Biomedical Sciences, Nagasaki, JPN; 2 Rehabilitation, Oita Oka Hospital, Oita, JPN; 3 Rehabilitation, Juko Memorial Nagasaki Hospital, Nagasaki, JPN; 4 Physical Therapy Science, Nagasaki University Graduate School of Biomedical Sciences, Nagasaki, JPN; 5 Health Sciences, Graduate School of Health Sciences, Kumamoto Health Science University, Kumamoto, JPN

**Keywords:** cardiopulmonary exercise test, rate of perceived exertion, virtual reality cycling, psychophysiology, virtual reality rehabilitation, visual flow velocity, virtual reality

## Abstract

Introduction: Virtual reality cycling (VRC) is simulated outdoor cycling with changes in scenery in virtual reality (VR) with rotating ergometer pedals. The speed at which the scenery changes, which is the visual flow velocity, can shift according to the same pedal rotation speed.

Objectives: This study investigated the effects of different visual flow velocities on the psychophysiological responses of cyclists using the VRC.

Methods: Participants were asked to cycle for 20 min at 30% of their maximum exercise load under four conditions: (1) bicycle ergometer without VR (control), (2) VRC at normal visual flow velocity (VRC-normal), (3) VRC at 0.5 times the visual flow velocity of VRC-normal (VRC-slow), and (4) VRC at 1.5 times the visual flow velocity of VRC-normal (VRC-fast). The order of the four conditions was randomized in a counterbalanced design. The heart rate and rating of perceived exertion were recorded during the exercise. Participants graded their enjoyment of the task using the physical activity enjoyment scale (PACES). The measured data were analyzed by comparing the visual flow velocity conditions (VRC-slow, VRC-normal, and VRC-fast), and comparing the VRC and bicycle ergometer (VRC-normal and control).

Results: A total of 24 participants were enrolled in the study. There was a significant main effect observed in the PACES score (F_(2,46)_=20.129, p<0.001, partial η^2^=0.467). In the post-hoc test for the PACES, significant differences were found in the following combinations: VRC-normal > VRC-slow (p=0.005); VRC-fast > VRC-normal (p=0.003); and VRC-fast > VRC-slow (p<0.001). In the modified Borg scale for lower-limb fatigue, there were significant differences in time factor (F_(2,46)_=134.048, p<0.001, partial η^2^=0.854) and interaction effects (F_(4,92)_=3.156, p=0.018, partial η^2^=0.121). In the post-hoc test for the modified Borg scale, significant trends were found in the following combinations: VRC-normal > VRC-fast (p=0.068) and VRC-slow > VRC-fast (p=0.083).

Conclusion: The results suggest that a slower visual flow velocity may reduce the enjoyment of exercise, whereas a faster visual flow velocity may make the exercise feel less fatigued and more enjoyable.

## Introduction

Exercise is the most widely used form of rehabilitation. Exercise has the advantage of being beneficial to physical and mental health [1]. However, the problem of being tired and not enjoying the activity leads to the discontinuation of exercise. These problems are particularly true for patients and prevent therapists from fully utilizing rehabilitation with good patient adherence [2]. To solve this problem, it is clinically important to identify less fatigue and enjoyable exercise methods.

In recent years, there has been a rapid increase in research and software development related to virtual reality (VR) rehabilitation, which combines VR technology and exercise [3-6]. There are two types of VR, namely non-immersive and immersive. Non-immersive VR is the experience of a virtual world on a computer screen without being fully immersed, while immersive VR is a physically immersive experience in a virtual world that involves wearing head-mounted displays (HMD). VR rehabilitation mainly uses immersive VR to allow subjects to touch, feel, and manipulate 3D objects in the virtual world and access multisensory functions to simulate performance beyond their capabilities. VR may also be effective in improving exercise adherence due to its gaming elements, which provide a sense of enjoyment and satisfaction.

The virtual reality cycling (VRC) program is a type of VR rehabilitation, which simulates outdoor cycling in a virtual world by changing the cycling scenery projected onto the HMD according to the rotation of the ergometer pedals. In recent years, there has been an increasing number of studies on the VRC, which has been reported to cause less fatigue and more enjoyment and self-efficacy in subjects than bicycle ergometers [7-12]. Longitudinal studies have also reported increased exercise participation and fitness with the VRC than with bicycle ergometers, suggesting that the VRC may be a way to solve exercise problems [12].

We focused on the visual flow velocity as a factor that might make the VRC more effective. Stationary observers perceive self-motion when a moving visual stimulus is presented in their visual field. This illusory self-motion is termed vection and visual stimuli are termed optical flow [13,14]. In the VRC, the cycling scenery forms an optical flow in the subject's vision, even when the subject is exercising on a stationary ergometer, providing an experience of cycling in a virtual world. The velocity of the optical flow is called the visual flow velocity, which is an important cue for the perception of movement velocity.

However, the effects of the different visual flow velocities on psychophysiological responses during the VRC remain unclear. The only previous study done has found no significant effects on any physiological response or fatigue due to different visual flow velocities during the VRC. However, visual flow velocity significantly influenced participant ratings of vitality and pleasure, wherein faster visual flow velocity resulted in a more positive mood state [15]. The limitations of the previous study included the following: the subjects were all male, the relative unification of exercise load between subjects was not possible, and the cycling scenery of the VRC was not synchronized with pedal rotation. Hence, to solve these protocol problems, the purpose of this study was to examine and compare the effects of different visual flow velocities on psychophysiological responses, such as fatigue, heart rate, and enjoyment of exercise, during the VRC under conditions of the relative uniformity of the participants’ exercise load. We hypothesized that a faster visual flow velocity would be more exhilarating, enjoyable, and less fatiguing due to the illusion of moving faster, whereas a slower visual flow velocity would be more boring and fatiguing due to the illusion of moving slower. We based our hypothesis on the phenomenon of pseudo-haptics. Pseudo-haptics is a phenomenon in which users experience haptic feedback by observing a visual stimulus that is designed to be distorted based on user input [16]. Previous study on pseudo-haptics has shown that, based on the fact that lighter (heavier) objects are easier (harder) to move, manipulating the position of the user's hand in VR can increase or decrease the movement of the displayed hand and induce the illusion of weight [17]. In addition, since only a few studies have compared VRC and bicycle ergometers under a relatively uniform exercise load, the secondary purpose of this study was to compare psychophysiological responses between the VRC and bicycle ergometers.

## Materials and methods

Participants

The participants were healthy young adults who were recruited through advertisements on university campuses. The inclusion criteria were healthy young adults aged 18-25 years who did not have VR sickness by VRC and who were able to speak, read, and write Japanese. Participants experienced VRC before the experiment and were considered not to have VR sickness if their Simulator Sickness Questionnaire (SSQ) score was less than 19 points [18,19]. The exclusion criteria were vehicle sickness, cardiovascular disorders, respiratory disorders, musculoskeletal disorders, and psychological disorders. This study was carried out according to the Declaration of Helsinki and was approved by the Ethics Committee of Nagasaki University Graduate School of Biomedical Sciences (approval number: 23071304-2; date of approval: 28 July 2023). All participants provided informed written consent before participating.

The sample size was calculated using G*Power version 3.1.9.6. software (Dusseldorf, Germany). A priori power analysis determined that with a power of 0.80 and alpha ≤ 0.05 on a repeated-measures one-way analysis of variance, 24 participants were required to detect a significant difference in the post-exercise physical activity enjoyment scale (PACES) scores with moderate effect size (f=0.25). Thus, a target sample size of 26-28 participants was planned to allow for the possibility of participants dropping out of the study (approximately 20%).

Methods

**Figure 1:**
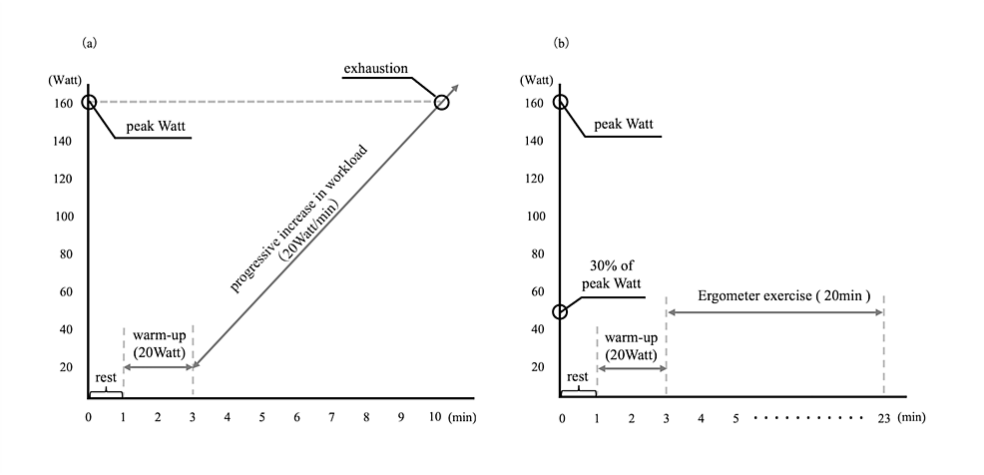
(a) The exercise loading protocol for the CPET and (b) the exercise loading protocol in ergometer exercise (second to fifth sessions) (a) The exercise loading protocol for the CPET, with the vertical axis showing the exercise load (watt) and the horizontal axis showing the time (min). CPET was performed using a bicycle ergometer to measure the maximal exercise load (peak watt) of the participants and consisted of 1 min rest and 2 min 20 watt pedaling at 50 revolutions per minute, followed by a progressive increase in the workload of 20 watt/min until exhaustion. (b) The exercise loading protocol in ergometer exercise (second to fifth sessions). In the second to fifth sessions, ergometer exercises were performed for 20 min each under four exercise conditions at 30% of peak watt. CPET, cardiopulmonary exercise test

This was an experimental crossover study wherein the participants participated in five sessions separated by at least 24 h. In the first session, cardiopulmonary exercise testing (CPET) was performed to measure maximal exercise load (peak watt) (Figure 1a). In the second to fifth sessions, ergometer exercises were performed for 20 min each under four exercise conditions at 30% of peak watt (Figure 1b). The exercise conditions were the following: (1) bicycle ergometer without VR (control), (2) VRC at normal visual flow velocity (VRC-normal), (3) VRC at 0.5 times the visual flow velocity of VRC-normal (VRC-slow), and (4) VRC at 1.5 times the visual flow velocity of VRC-normal (VRC-fast). The VRC-normal is the same as that of general outdoor cycling and is the default setting in the VRC application (VZfit, VIRzoom, USA). The experimental order of sessions two to five was counterbalanced by block randomization using the envelope method. If malaise, fatigue, or myalgia were present before each session, the session was performed on a different day once the symptoms had disappeared.

CPET

The CPET is an objective assessment of exercise capacity performed to ensure the relative unification of exercise load between participants [20]. CPET was performed using a bicycle ergometer (232CXL, COMBI, Japan) to measure the peak watts of the participants, and consisted of 1 min rest and 2 min 20 watt pedaling at 50 revolutions per minute, followed by a progressive increase in the workload of 20 watt/min until exhaustion (Figure 1a).

VRC system

**Figure 2:**
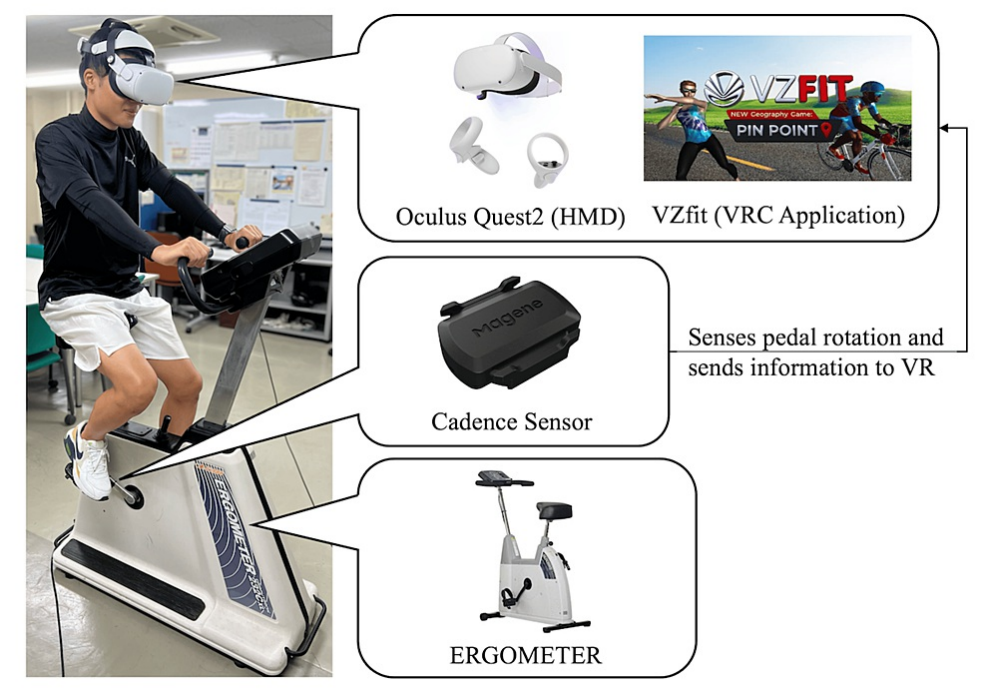
The VRC system The VRC system consisted of an HMD (Meta Quest2, Oculus, USA), VRC application (VZfit, VIRzoom, USA), bicycle ergometer (232CXL9, COMBI, Japan), and cadence sensor (Cadence & Velocity Dual Mode Sensor, Qingdao Magene Intelligent Technology, China). HMD, head-mounted display; VRC, virtual reality cycling

The VRC system consisted of an HMD (Meta Quest2, Oculus, USA), VRC application, bicycle ergometer (232CXL9, COMBI, Japan), and cadence sensor (Cadence & Velocity Dual Mode Sensor, Qingdao Magene Intelligent Technology, China) (Figure 2). The VRC simulates outdoor cycling by displaying a changing scenery on the HMD, based on the number of revolutions detected by the cadence sensor attached to the ergometer pedals. The cycling course for the VRC was the "French Country side-Day" of the "Virtual Ride" in the VRC application, which is a cycling course in a natural daytime environment with forests, houses, and a random mix of hill and flat roads. The experiment was conducted on a course where the landscape was the same for all participants. The VRC application can be used to change the visual flow velocity.

Measurement

Participant Characteristics

Participant characteristics, such as their gender, age, height, weight, and body mass index, were recorded.

Psychophysiological Response

PACES: Exercise enjoyment was measured using the PACES, which is an 18-item questionnaire with a seven-point bipolar Likert scale with points awarded from one to seven depending on the answer chosen (minimum of 18 points and maximum of 126 points) (Table 3 of Appendix) [21]. Higher total scores indicated higher levels of enjoyment in physical activity. The reported Cronbach’s alpha for the scale was 0.96, and the scale was found to be reliable in adults [22,23].

Heart rate: The heart rate was monitored during the session with an Apple Watch SE (Apple, USA), and data were recorded every minute from the start to the end of the exercise.

Modified Borg scale: The perceived exertion of participants, such as the sensation of dyspnea and lower-limb fatigue, was recorded every minute using a modified Borg scale. The modified Borg scale is an index of perceived exertion by participants during exercise and is answered on a scale of 0 (easiest) to 10 (hardest) [24].

Statistical analyses

After confirming normal distributions using the Shapiro-Wilk test, the measured data were analyzed by comparing the visual flow velocity conditions (VRC-slow, VRC-normal, and VRC-fast) and comparing the VRC and bicycle ergometer (VRC-normal and control).

Comparison of Visual Flow Velocity Conditions (VRC-Slow, VRC-Normal, and VRC-Fast)

Since the PACES scores were normally distributed, a repeated-measures one-way analysis of variance was performed, and the Bonferroni method was used for the post-hoc tests. For the modified Borg scale and heart rate, a repeated-measures two-way analysis of variance was performed, and the Bonferroni method was used for post-hoc tests. The two factors used for the analyses were exercise condition (VRC-slow, VRC-normal, and VRC-fast) and exercise duration (1-3 min: warm-up, 4-13 min: first half of the exercise, and 14-23 min: second half of the exercise).

Comparison of the VRC and Bicycle Ergometer (VRC-Normal and Control)

The PACES data were analyzed using t-tests since these were normally distributed. For the modified Borg scale and heart rate, a repeated-measures two-way analysis of variance was performed, and the Bonferroni method was used for post-hoc tests. The two factors used for the analyses were exercise condition (VRC-normal and control) and exercise duration (1-3 min: warm-up, 4-13 min: first half of the exercise, and 14-23 min: second half of the exercise). 

Data were analyzed using the SPSS software version 29 (IBM Corp, Armonk, NY, USA). Significant differences were set at p<0.05. Significant trends were considered at p<0.1.

## Results

Participants characteristics

A total of 27 participants (11 men and 16 women) participated in the experiments, and three participants were excluded due to VR sickness. Participant characteristics are summarized in Table 1.

**Table 1:** Participants characteristics The data are mean ± SD. BMI, body mass index; IPAQ, International Physical Activity Questionnaire

Characteristics			Values
Age, y			21.3±1.3
Male sex, n(%)			11 (40)
Weight, kg			55.1±6.2
Height, cm			165.1±6.9
BMI, kg/m^2^			20.2±1.8
IPAQ			1834.9±1876.9
Peak watt, W			164±36.2

Comparison of visual flow velocity conditions (VRC-slow, VRC-normal, and VRC-fast)

**Figure 3:**
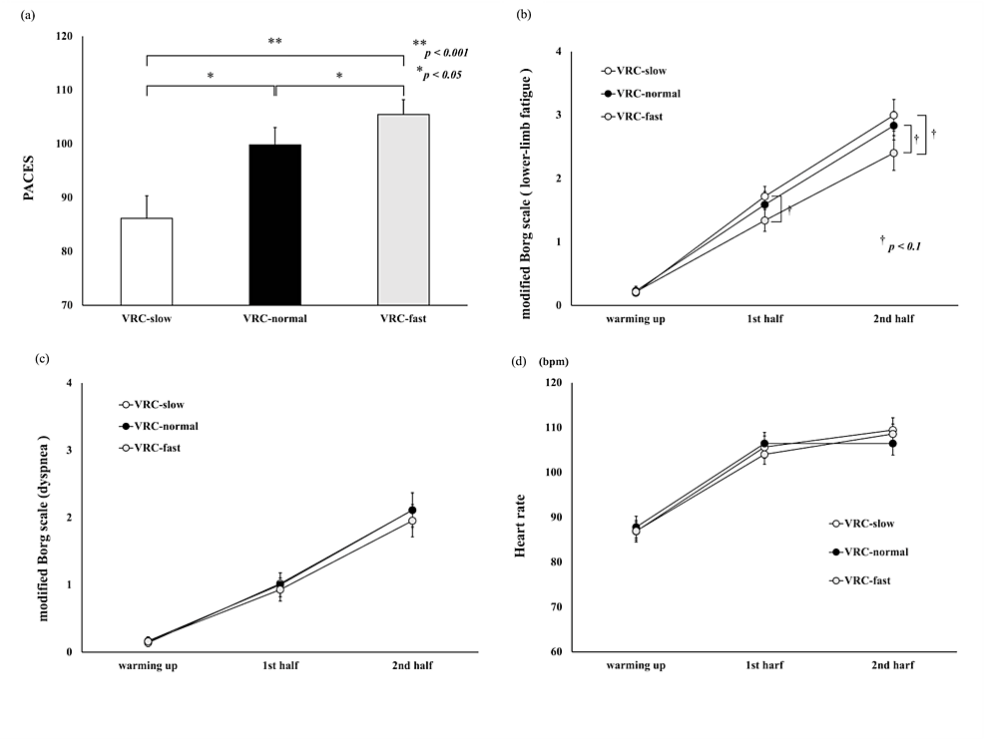
The comparison of visual flow velocity conditions (VRC-slow, VRC-normal, and VRC-fast) White indicates VRC-slow, black indicates VRC-normal, and grey indicates VRC-fast scores. (a) PACES score after the VRC. Data are shown as means (+SD). Significant differences were found in the following combinations: VRC-normal > VRC-slow (p=0.005), VRC-fast > VRC-normal (p=0.003), and VRC-fast > VRC-slow (p<0.001). (b) The modified Borg Scale (lower-limb fatigue) scores during the VRC. Data are shown as means (±SE). There were significant trends in the first (VRC-slow > VRC-fast, p=0.084) and second halves of the exercise (VRC-normal > VRC-fast, p=0.068; VRC-slow > VRC-fast, p=0.083). (c) The modified Borg Scale (dyspnea) scores during the VRC. Data are shown as means (±SE). (d) The heart rate results during the VRC. Data are shown as means (±SE). PACES, physical activity enjoyment scale; VRC, virtual reality cycling

There was a significant main effect observed in the PACES score (F_(2,46)_=20.129, p<0.001, partial η^2^=0.467). In the post-hoc test using the Bonferroni method for the PACES score, significant differences were found in the following combinations: VRC-normal > VRC-slow (p=0.005), VRC-fast > VRC-normal (p=0.003), and VRC-fast > VRC-slow (p<0.001) (Figure 3a).

The modified Borg scale and heart rate data were analyzed using a two-way analysis of variance. In the modified Borg scale (lower-limb fatigue), there were significant differences in the time factor (F_(2,46)_=134.048, p<0.001, partial η^2^=0.854) and interaction effects (F_(4,92)_=3.156, p=0.018, partial η^2^=0.121). There were significant trends in the visual flow velocity factor (F_(2,46)_=0.062, p=0.062, partial η^2^=0.114). In the post-hoc test using the Bonferroni method for the modified Borg scale (lower-limb fatigue), there were significant trends in the first (VRC-slow > VRC-fast, p=0.084) and second halves of the exercise (VRC-normal > VRC-fast, p=0.068; VRC-slow > VRC-fast, p=0.083) (Figure 3b). No significant differences were found in the modified Borg scale (dyspnea) or heart rate (Figures 3c, 3d).

Comparison of the VRC and bicycle ergometer (VRC-normal and control)

**Figure 4:**
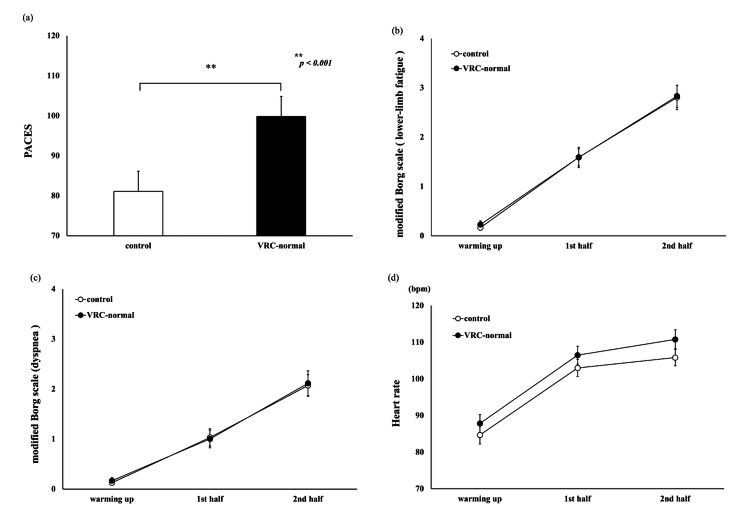
The comparison of the VRC and bicycle ergometer (VRC-normal and control) Black indicates VRC-normal and white indicates control. (a) PACES scores after VRC and bicycle ergometer. Data are shown as means (+SD). Significant differences were found between VRC-normal and control (p<0.001). (b) The modified Borg Scale (lower-limb fatigue) score during VRC and bicycle ergometer. Data are shown as means (±SE). (c) The modified Borg Scale (dyspnea) score during VRC and bicycle ergometer. Data are shown as means (±SE). (d) The heart rate scores during VRC and bicycle ergometer. Data are shown as means (±SE). PACES, physical activity enjoyment scale; VRC, virtual reality cycling

There was a significant main effect observed in the PACES score (t_(23)_=-4.09, p<0.001, d=-0.835) (Figure 4a). The modified Borg scale and heart rate data were analyzed using a two-way analysis of variance. No significant differences were found in the modified Borg scale (Figures 4b, 4c). In the heart rate data, there were significant differences in the time factor (F_(1,23)_=4.517, p=0.045, partial η^2^=0.164) and conditional factor (F_(2,46)_=343.901, p<0.001, partial η^2^=0.937) (Figure 4d). However, there were no significant differences in the interaction effects (F_(2,46)_=2.262, p=0.116, partial η^2^=0.09).

## Discussion

To the best of our knowledge, this is the first study to examine the effects of different visual flow velocities on psychophysiological responses, such as fatigue, heart rate, and enjoyment of exercise, during the VRC under conditions of the relative uniformity of the participants’ exercise load. In terms of exercise enjoyment, VRC-normal was significantly higher than VRC-slow, and VRC-fast was significantly higher than VRC-normal. For lower-limb fatigue, VRC-fast showed a significantly lower trend than VRC-slow in the first half of the exercise (1-10 min), and VRC-fast showed a significantly lower trend than VRC-normal and VRC-slow in the second half of the exercise (11-20 min). A comparison of the VRC and bicycle ergometer showed that the VRC was significantly higher than the bicycle ergometer for exercise enjoyment.

Effects of visual flow velocity on enjoyment

The results of this study showed that VRC-normal was significantly higher than VRC-slow, and VRC-fast was significantly higher than VRC-normal and VRC-slow in terms of exercise enjoyment. This result is identical to a previous study that showed that faster visual flow was associated with more positive self-reported vitality and pleasure, and this finding also complements the result of the previous study as a study in conditions where participants' exercise load was relatively uniform [15]. The VRC-fast may have led patients to experience a sense of exhilaration and enjoyment of exercise owing to the motion illusion of faster progress, whereas the VRC-slow may have led patients to experience a sense of boredom and decreased enjoyment of exercise owing to the motion illusion of less progress. Hence, setting the visual flow velocity of the VRC to at least faster than normal rather than slower may have a positive effect on the psychological response of patients.

Effects of visual flow velocity on lower-limb fatigue

The VRC-fast tended to be significantly lower than the VRC-slow in the first half of the exercise (1-10 min) and VRC-fast tended to be significantly lower than VRC-normal and VRC-slow in the second half of the exercise (11-20 min) in terms of lower-limb fatigue. These results differ from those of a previous study, which found that varying the visual flow velocity in the VRC did not affect the perceived exertion level of participants [15]. The differences between the protocols of the present study and those of the previous study are shown in Table 2 [15].

**Table 2:** Differences between the previous study and the present study

		Previous study				Present study			
Exercise time		5 min				20 min			
Exercise load		80 W				30% of peak watt			
Experimental design		Three experiments conducted in one day				One experiment per day conducted			
Borg scale		Borg scale (perceived exertion)				Modified Borg scale (dyspnea and lower-limb fatigue)			
VRC system		Smartphone-mounted VR (PVRGBT01BK, Elecom, Osaka, Japan) displayed riverbed cycling scenes from a flat road				VRC application (VZfit, VIRzoom, USA)			

Fatigue during exercise is perceived by subjects through the integration and feedback of physical stress and psychophysiological information. In the present study, the physical stress affecting fatigue was considered to be at the same level because the exercise load was the same. A possible reason for the differences in lower-limb fatigue under identical physical stress conditions is that the different visual flow velocities of the VRC may have produced pseudo-haptics. Pseudo-haptics is a phenomenon in which users experience haptic feedback by observing a visual stimulus designed to be distorted based on user input [17]. Therefore, a higher visual flow velocity may have resulted in the illusion of a lower pedal load and lower-limb fatigue. The reason why there was no difference in dyspnea was considered to be that the exercise load was low, and the exercise was not of such load that breathing became difficult. These results suggest that a faster visual flow velocity may result in a lower perception of lower-limb fatigue, such that exercise may be perceived as easier.

Effects of the use of VR

The VRC has been shown to make exercise more enjoyable than a bicycle ergometer. This result is identical to the results of several previous studies that reported higher exercise enjoyment with the VRC than with a bicycle ergometer, and the enjoyment provided by the game elements of VR was considered to have a significant impact [7,11,25,26]. It has been suggested that under the same exercise load conditions, the VRC may be more effective for exercise promotion and rehabilitation because exercise is more enjoyable than on a bicycle ergometer.

Limitation

This study had two limitations. First, the exercise load settings were low; medium and high exercise loads were not studied. Second, the visual flow velocity was only examined for conditions 0.5 times slower than the normal velocity and 1.5 times faster than the normal velocity. In the future, it will be necessary to examine the extent and most optimal exercise load and visual flow velocity settings that are most effective for psychophysiological responses.

Clinical implication

Aerobic exercises, such as ergometers performed in hospital facilities, tend to be monotonous activities because they are performed indoors, and the surrounding scenery does not change. Therefore, patients may experience difficulty in doing these unenjoyable activities; hence, adherence to rehabilitation may be difficult to establish. However, the results of this study suggest that the VRC has the potential to provide rehabilitation with good adherence because it is a more enjoyable exercise than a bicycle ergometer and can be applied to VR techniques, such as visual flow velocity. Furthermore, the VZfit is linked to Google Street View; therefore, it is possible to cycle while viewing scenery worldwide. This technology can simulate VRC trips to places visited in the past and could be offered to patients with dementia as a reminiscence method to approach cognitive function. In addition, the commercially available VRC system used in this study is inexpensive and versatile in clinical practice. However, the current technology cannot be used in all cases because of the possibility of VR sickness. In the future, it will be necessary to further improve VR technology and examine the effectiveness of the VRC in clinical applications.

## Conclusions

The results of this study suggest that slower visual flow velocity may reduce the enjoyment of exercise, whereas faster visual flow velocity may make the exercise feel more exciting and enjoyable. Therefore, by applying modifications to the visual flow velocity of the VRC during rehabilitation, it may be possible to adjust the enjoyment and fatigue experienced by the subject for the same exercise load. It was also suggested that VR made the exercises more enjoyable; hence, VR with game elements should be recommended in clinical practice to make exercise enjoyable.

## Appendices

**Table 3:** PACES PACES is an 18-item questionnaire with a seven-point bipolar Likert scale with points awarded from one to seven depending on the answer chosen (minimum of 18 points and maximum of 126 points). Higher total scores indicated higher levels of enjoyment in physical activity. PACES, physical activity enjoyment scale

PACES									
		1	2	3	4	5	6	7	
1	I enjoy it.								I hate it
2	I feel bored.								I feel interested.
3	I dislike it.								I like it.
4	I find it pleasurable.								I find it unpleasurable.
5	I am very absorbed in this activity.								I am not at all absorbed in this activity.
6	It’s no fun at all.								It's a lot of fun.
7	I find it energizing.								I find it tiring.
8	It makes me depressed.								It makes me happy.
9	It’s very pleasant.								It's very unpleasant.
10	I feel good physically while doing it.								I feel bad physically while doing it.
11	It’s very invigorating.								It's not at all invigorating.
12	I am very frustrated by it.								I am not at all frustrated by it.
13	It’s very gratifying.								It's not at all gratifying.
14	It's very exhilarating.								It's not at all exhilarating.
15	It's not at all stimulating.								It's very stimulating.
16	It gives me a strong sense of accomplishment.								It does not give me any sense of accomplishment.
17	It's very refreshing.								It's not at all refreshing.
18	I felt as though I would rather be doing something else.								I felt as though there was nothing else I would rather be doing.
